# Evolution of Sepsis Management in the Emergency Department: From Early Goal-Directed Therapy to Personalized Resuscitation

**DOI:** 10.7759/cureus.112866

**Published:** 2026-07-17

**Authors:** Naser Mohamad Mansoor, Lan Ghassan, Ali M Haider Ali, Hussain AlShehabi, Fatema Mohamed

**Affiliations:** 1 Accident and Emergency, Salmaniya Medical Complex, Manama, BHR; 2 Medicine, Royal College of Surgeons in Ireland, Muharraq, BHR; 3 Pediatric Emergency Medicine, Salmaniya Medical Complex, Manama, BHR

**Keywords:** early recognition of sepsis, sepsis and shock, sepsis protocols, sepsis related research and publications, sepsis screen

## Abstract

Sepsis remains a leading cause of morbidity and mortality worldwide and represents a major challenge in emergency medicine. Early recognition and timely intervention are critical determinants of patient outcomes. The introduction of early goal-directed therapy (EGDT) in 2001 transformed sepsis management by promoting protocolized hemodynamic resuscitation during the initial hours of care. However, subsequent evidence challenged the routine use of invasive EGDT targets and led to significant changes in contemporary sepsis management strategies. This narrative review examines the evolution of sepsis management in the emergency department, focusing on the development, implementation, and reassessment of EGDT. Literature published between 2001 and 2026 was reviewed using major medical databases and guideline repositories, with emphasis on landmark randomized controlled trials (RCTs), systematic reviews, meta-analyses, and international guideline statements. Key studies, including Protocolized Care for Early Septic Shock (ProCESS), Australasian Resuscitation in Sepsis Evaluation (ARISE), and Protocolised Management in Sepsis (ProMISe), demonstrated no mortality benefit of rigid protocolized EGDT compared with contemporary standard care, contributing to a shift toward individualized, physiology-guided resuscitation strategies. Current evidence supporting early antimicrobial administration, balanced crystalloid resuscitation, dynamic assessment of fluid responsiveness, early vasopressor initiation, and lactate-guided monitoring is reviewed, along with ongoing controversies involving antibiotic timing, fluid management, biomarker-guided therapy, and personalized sepsis care. Although protocolized EGDT is no longer routinely recommended, its emphasis on early recognition and prompt intervention continues to influence modern sepsis management. Contemporary practice favors individualized, patient-centered approaches focused on rapid diagnosis, timely antimicrobial therapy, targeted hemodynamic support, and continuous reassessment of tissue perfusion, while future advances are likely to involve biomarker-guided treatment, precision medicine, and artificial intelligence-assisted clinical decision support.

## Introduction and background

Sepsis is a major cause of morbidity and mortality worldwide and represents a substantial burden on healthcare systems. It is defined as a life-threatening organ dysfunction caused by a dysregulated host response to infection, with organ dysfunction identified by an increase of 2 or more points in the Sequential Organ Failure Assessment (SOFA) score in the setting of suspected or confirmed infection [1]. Septic shock represents a more severe subset characterized by profound circulatory and metabolic abnormalities, defined by persistent hypotension requiring vasopressor therapy to maintain a mean arterial pressure (MAP) ≥65 mmHg together with serum lactate levels >2 mmol/L despite adequate fluid resuscitation. Mortality among patients with septic shock frequently exceeds 40%, emphasizing the importance of prompt recognition and intervention [2,3].

The emergency department (ED) plays a critical role in sepsis management because early recognition and therapeutic intervention strongly influence clinical outcomes. Delays in diagnosis, antibiotic administration, and hemodynamic resuscitation have consistently been associated with increased mortality, progression to multiorgan dysfunction, and prolonged hospitalization [3,4]. Consequently, considerable efforts have focused on developing strategies that facilitate rapid identification and timely intervention in patients with sepsis.

A major turning point in sepsis management occurred with the introduction of early goal-directed therapy (EGDT) by Rivers and colleagues in 2001, which demonstrated a significant reduction in mortality through protocolized early resuscitation targeting specific hemodynamic parameters [5]. This approach established the foundation for structured sepsis bundles and substantially influenced international management guidelines. However, subsequent large multicenter trials, including ProCESS, ARISE, and ProMISe, failed to demonstrate additional mortality benefit compared with contemporary standard care [2,6,7].

These findings prompted a transition from rigid protocol-based strategies toward more individualized and physiology-guided approaches to sepsis resuscitation. The evolution of sepsis definitions and diagnostic criteria is summarized in Table 1. Contemporary management increasingly emphasizes patient-specific assessment, dynamic hemodynamic monitoring, antimicrobial stewardship, and early targeted intervention. This review aims to examine the evolution of sepsis management in the ED, with particular emphasis on the development, reassessment, and ongoing influence of EGDT, as well as contemporary evidence shaping current clinical practice.

**Table 1:** Evolution of Sepsis Definitions SIRS: systemic inflammatory response syndrome, SOFA: Sequential Organ Failure Assessment, qSOFA: quick Sequential Organ Failure Assessment, MAP: mean arterial pressure. Adapted from references [1,3,5].

Feature	Sepsis-1/Sepsis-2	Sepsis-3
Year introduced	1991, revised 2001	2016
Definition of sepsis	Infection + ≥2 SIRS criteria	Life-threatening organ dysfunction caused by a dysregulated host response to infection
Organ dysfunction assessment	Not required for diagnosis	SOFA score increase ≥2 points
Severe sepsis	Sepsis + organ dysfunction	Eliminated (considered redundant)
Septic shock	Sepsis-induced hypotension despite fluids	Vasopressor requirement to maintain MAP ≥65 mmHg and lactate >2 mmol/L despite adequate fluid resuscitation
Screening tool	SIRS	qSOFA (prognostic tool)
Major limitation addressed	Low specificity of SIRS	Improved identification of high-risk patients

## Review

Methods

A narrative literature review was conducted to examine the evolution of sepsis management in the ED, with particular emphasis on EGDT, contemporary resuscitation strategies, and recent guideline developments. The final literature search was conducted on July 12, 2026.

Relevant literature published between January 2001 and July 2026 was identified through searches of PubMed/MEDLINE, Google Scholar, and major guideline repositories. Search terms included combinations of "sepsis," "septic shock," "early goal-directed therapy," "emergency department," "Surviving Sepsis Campaign," "fluid resuscitation," "vasopressors," "lactate clearance," "biomarkers," and "personalized sepsis care."

Priority was given to landmark randomized controlled trials, systematic reviews, meta-analyses, and international guideline statements that have shaped contemporary sepsis management. Additional literature addressing Gulf region epidemiology, antimicrobial resistance, and regional considerations was included where available.

Pathophysiology of sepsis

Sepsis is a complex syndrome resulting from a dysregulated host response to infection that culminates in life-threatening organ dysfunction. According to Sepsis-3, sepsis is characterized by life-threatening organ dysfunction resulting from a dysregulated host response to infection rather than from microbial burden alone, emphasizing the central role of host-pathogen interactions in disease progression [1,8]. This distinction is important because morbidity and mortality are influenced not only by microbial burden but also by the host’s systemic inflammatory and immunologic response. The Sepsis-3 framework further recognizes that sepsis represents a heterogeneous syndrome influenced by multiple host factors, including age, comorbidities, genetic determinants, and environmental factors, all of which may alter clinical presentation and disease progression. Sepsis remains a major global health burden and continues to be associated with substantial morbidity and mortality worldwide [1,8].

The pathophysiological process begins with activation of the innate immune system following recognition of invading pathogens through pathogen-associated molecular patterns (PAMPs) and damage-associated molecular patterns (DAMPs). These molecular signals are detected by pattern-recognition receptors, particularly toll-like receptors (TLRs) expressed on macrophages, neutrophils, and endothelial cells. Activation of these receptors initiates intracellular signaling pathways that stimulate the release of pro-inflammatory mediators, including tumor necrosis factor-alpha (TNF-α), interleukin-1β (IL-1β), and interleukin-6 (IL-6). The resulting inflammatory response promotes widespread vasodilation, increased vascular permeability, and recruitment of inflammatory cells, contributing to the systemic manifestations commonly associated with sepsis [8,9].

Simultaneously, sepsis induces a compensatory anti-inflammatory response mediated by cytokines such as interleukin-10 and by other immunosuppressive mechanisms. Although initially protective, excessive activation of these pathways may result in immune dysfunction or immune paralysis, increasing susceptibility to secondary infections and poorer clinical outcomes [8]. Consequently, sepsis should not be viewed solely as a hyperinflammatory process but rather as a complex interaction between competing pro-inflammatory and anti-inflammatory responses.

Endothelial dysfunction represents a central component of sepsis pathophysiology. Under physiological conditions, the vascular endothelium maintains barrier integrity, regulates vascular tone, and modulates coagulation pathways. During sepsis, inflammatory mediators disrupt endothelial integrity, leading to capillary leakage, interstitial edema, and intravascular volume depletion. Endothelial activation also promotes a prothrombotic state through increased tissue factor expression and suppression of endogenous anticoagulant pathways, resulting in microvascular thrombosis. These disturbances impair microcirculatory blood flow and oxygen delivery at the tissue level, even when systemic hemodynamic parameters appear preserved [9].

At the cellular level, sepsis is characterized by mitochondrial dysfunction and metabolic derangements. Impaired oxidative phosphorylation reduces adenosine triphosphate (ATP) production, leading to cellular energy failure and progressive organ dysfunction. Concurrently, increased anaerobic metabolism contributes to lactate accumulation, reflecting both tissue hypoperfusion and impaired cellular oxygen utilization. Elevated serum lactate, therefore, serves as both an indicator of disease severity and an important prognostic marker in emergency settings [8,9]. In addition, widespread apoptosis of immune and parenchymal cells further contributes to tissue injury and progression toward multiorgan dysfunction [8].

As these pathologic processes progress, sepsis may evolve into septic shock, the most severe manifestation of the syndrome. Consistent with Sepsis-3, septic shock is clinically defined by persistent hypotension requiring vasopressor therapy to maintain MAP ≥65 mmHg together with serum lactate levels >2 mmol/L despite adequate fluid resuscitation [1,8]. The underlying hemodynamic profile is characterized by profound vasodilation, increased vascular permeability, relative hypovolemia, and myocardial dysfunction, all of which contribute to impaired tissue perfusion and oxygen delivery. These abnormalities are associated with substantially increased mortality risk [8,9].

Disease progression may ultimately result in multiple organ dysfunction syndrome (MODS). Pulmonary manifestations frequently include acute respiratory distress syndrome, resulting from diffuse alveolar-capillary membrane injury and impaired gas exchange. Renal involvement commonly presents as acute kidney injury secondary to hypoperfusion, inflammation, and microvascular thrombosis. Cardiovascular compromise may include persistent hypotension and sepsis-induced myocardial dysfunction, whereas neurological manifestations range from delirium to sepsis-associated encephalopathy. Hepatic dysfunction, coagulopathy, and disseminated intravascular coagulation may also occur in advanced disease states [8,9].

Common sources of sepsis include pneumonia, urinary tract infections, and intra-abdominal infections, each associated with distinct microbial patterns and clinical implications. Early identification of the infectious source is essential because it guides empiric antimicrobial therapy and source-control interventions, both of which significantly influence patient outcomes [8,9].

Overall, sepsis pathophysiology reflects a complex interplay between immune dysregulation, endothelial injury, microcirculatory dysfunction, and metabolic failure. The progression from localized infection to septic shock and multiple organ dysfunction underscores the importance of early recognition and prompt intervention, as treatment delays remain strongly associated with adverse outcomes [8,9].

Early goal-directed therapy

EGDT was introduced by Rivers and colleagues in 2001 in an effort to reduce the high mortality associated with severe sepsis and septic shock through standardized resuscitation during the earliest phase of care. In the landmark randomized controlled trial conducted in an urban ED, adult patients presenting with severe sepsis or septic shock were randomized to receive either six hours of protocolized resuscitation or conventional therapy before admission to the intensive care unit. The EGDT approach was based on the principle that early hemodynamic optimization, before the development of irreversible organ injury, could correct the imbalance between systemic oxygen delivery and oxygen demand that contributes to tissue hypoxia and progressive multisystem dysfunction in sepsis [5].

The protocol established specific physiologic targets to guide resuscitation, as summarized in Table 2. Initial management involved rapid intravenous fluid administration to achieve a central venous pressure (CVP) of 8-12 mmHg, thereby optimizing preload. In patients with persistent hypotension despite fluid resuscitation, vasopressors were administered to maintain a MAP ≥65 mmHg and preserve organ perfusion. Central venous oxygen saturation (ScvO₂) was used as a surrogate marker of the balance between oxygen delivery and consumption, with a target ScvO₂ ≥70%. If ScvO₂ remained below target values, blood transfusions were administered to achieve a hematocrit ≥30%, followed by dobutamine infusion when necessary to augment cardiac output and improve tissue oxygen delivery [5]. Overall, the protocol integrated aggressive fluid administration, vasoactive support, transfusion strategies, and inotropic therapy into a structured resuscitation pathway that was repeatedly reassessed during the initial six-hour treatment period.

**Table 2:** Key Components of Original Early Goal-Directed Therapy EGDT: early goal-directed therapy, CVP: central venous pressure, MAP: mean arterial pressure, ScvO₂: central venous oxygen saturation. Adapted from Rivers et al. [5].

EGDT Target	Goal
Central venous pressure (CVP)	8–12 mmHg
Mean arterial pressure (MAP)	≥65 mmHg
Urine output	≥0.5 mL/kg/hour
Central venous oxygen saturation (ScvO₂)	≥70%
Hematocrit	≥30% if ScvO₂ remained low
Dobutamine use	If ScvO₂ remained <70% despite fluids and transfusion
Duration of protocol	First 6 hours of resuscitation

The Rivers trial demonstrated a significant survival benefit associated with EGDT, with in-hospital mortality decreasing from 46.5% in the standard therapy group to 30% in the EGDT group, representing an absolute risk reduction of 16.5% and a number needed to treat of six [5]. Patients managed with EGDT achieved higher ScvO₂ values and lower lactate and base deficit levels over time, together with reduced severity of organ dysfunction as measured by the Acute Physiology and Chronic Health Evaluation II (APACHE II) and Simplified Acute Physiology Score II (SAPS II), and MODS scores. These findings suggested that early restoration of hemodynamic stability could attenuate the progression of global tissue hypoxia and subsequent organ dysfunction.

The concepts underlying EGDT were subsequently incorporated into international clinical practice guidelines. The 2012 Surviving Sepsis Campaign (SSC) guidelines recommended early quantitative resuscitation in patients with severe sepsis and septic shock, emphasizing interventions during the first six hours following diagnosis, including fluid resuscitation, vasopressor support, and monitoring of physiologic parameters such as CVP and ScvO₂ [10]. Although evidence quality varied across recommendations, these guidelines incorporated EGDT-related targets within a structured evidence-based framework for hemodynamic management. The subsequent publication of the Sepsis-3 definitions further influenced sepsis management by shifting emphasis away from protocolized treatment of broad inflammatory syndromes toward identification of patients with infection-associated organ dysfunction, thereby supporting more individualized approaches to resuscitation and risk stratification [1].

EGDT represented a major paradigm shift in sepsis care by emphasizing early, protocolized intervention based on measurable physiologic targets rather than relying solely on clinical judgment. Its focus on optimizing oxygen delivery and tissue perfusion during the initial hours of presentation established the foundation for subsequent sepsis management bundles and influenced the development of contemporary time-sensitive resuscitation strategies. Although initially designed for ED implementation, subsequent adaptations allowed its application across intensive care and other hospital settings, demonstrating the broader impact and flexibility of the EGDT model [11].

Major trials challenging EGDT

The early promise of EGDT was subsequently evaluated in a series of large multicenter randomized controlled trials conducted more than a decade after the original study [5]. The major trials and their principal findings are summarized in Table 3. These studies sought to determine whether protocolized hemodynamic targets continued to provide additional benefit within the context of evolving standards of sepsis care.

**Table 3:** Major Trials Evaluating Early Goal-Directed Therapy EGDT: early goal-directed therapy, ProCESS: Protocolized Care for Early Septic Shock, ARISE: Australasian Resuscitation in Sepsis Evaluation, ProMISe: Protocolised Management in Sepsis.

Trial	Year	Sample Size	Intervention	Primary Outcome	Key Finding
Rivers et al. [5]	2001	263	EGDT vs standard care	In-hospital mortality	Mortality reduced from 46.5% to 30%
ProCESS [2]	2014	1,341	EGDT vs protocolized standard therapy vs usual care	60-day mortality	No significant difference in mortality
ARISE [6]	2014	1,600	EGDT vs usual care	90-day mortality	No mortality benefit
ProMISe [7]	2015	1,260	EGDT vs usual care	90-day mortality	No mortality benefit
Overall Conclusion	—	4,464	—	—	Protocolized EGDT did not improve outcomes compared with contemporary usual care.

The Protocolized Care for Early Septic Shock (ProCESS) trial, conducted in the United States, enrolled 1,341 patients with early septic shock across 31 academic centers and compared three treatment strategies: protocol-based EGDT incorporating CVP and ScvO₂ monitoring, a less invasive protocolized standard therapy, and usual care directed by the treating clinical team [2]. Both protocol-based groups demonstrated greater use of central venous catheterization, intravenous fluids, vasopressors, dobutamine, and packed red blood cell transfusions, with adherence rates exceeding 88% in both intervention arms. Despite differences in treatment intensity, there was no significant difference in 60-day mortality among the groups (21.0% EGDT vs. 18.2% standard therapy vs. 18.9% usual care; P = 0.83), nor were differences observed in 90-day or one-year mortality. Secondary outcomes, including duration of organ support, incidence of acute renal failure, and intensive care unit (ICU) length of stay, were similarly comparable, although EGDT was associated with slightly higher ICU admission rates and transient increases in blood pressure [2].

Comparable findings were observed in the Australasian Resuscitation in Sepsis Evaluation (ARISE) trial, which enrolled 1,600 patients across 51 EDs in Australia, New Zealand, and selected centers in Europe and Hong Kong [6]. Patients were randomized to receive EGDT or usual care, with 90-day all-cause mortality as the primary outcome. Patients in the EGDT group received more intravenous fluids, vasopressors, red blood cell transfusions, and ScvO₂ monitoring during the initial six-hour period. However, despite the greater intensity of intervention, no significant difference in 90-day mortality was identified (18.6% EGDT vs. 18.8% usual care; absolute risk difference −0.3%; 95% CI −4.1 to 3.6; P=0.90). Secondary outcomes, including ICU length of stay, duration of organ support, and adverse events, were likewise similar between groups [6]. These findings suggested that protocolized targets such as CVP and ScvO₂ offered limited additional benefit when early recognition and timely resuscitation had already become integrated into routine clinical practice.

The Protocolised Management in Sepsis (ProMISe) trial, conducted in the United Kingdom, enrolled 1,260 patients across 56 National Health Service hospitals, with 630 patients assigned to EGDT and 630 assigned to usual care [7]. The primary endpoint was 90-day all-cause mortality. Patients receiving EGDT underwent more intensive interventions, including greater use of intravenous fluids, vasoactive medications, and red blood cell transfusions during the six-hour protocol period. This increased intervention intensity was associated with higher SOFA scores, more days of advanced cardiovascular support, and longer ICU stays. Nevertheless, mortality remained comparable between groups (29.5% EGDT vs. 29.2% usual care; relative risk 1.01; 95% CI 0.85-1.20; P = 0.90). Additional outcomes, including health-related quality of life and serious adverse events, also showed no significant differences. Furthermore, cost-effectiveness analyses demonstrated increased healthcare expenditures without corresponding improvements in quality-adjusted life years (QALYs), with an estimated probability of cost-effectiveness below 20% [7].

Collectively, these trials demonstrated that rigid protocolized EGDT does not provide a survival advantage over contemporary usual care [2,6,7]. Differences between outcomes observed in the original Rivers study and those reported in these later trials likely reflect substantial changes in sepsis management over time, including earlier administration of antibiotics, more rapid fluid resuscitation, and improved recognition of sepsis within EDs [3,5,10,11]. Moreover, the ProCESS, ARISE, and ProMISe studies showed that although protocolized EGDT increased the use of invasive monitoring and therapeutic interventions, these measures did not translate into improved patient survival [2,6,7].

Differences in baseline patient characteristics may also explain some of the observed variation in outcomes. Compared with the Rivers study population, patients enrolled in ProCESS, ARISE, and ProMISe generally exhibited lower illness severity, with many demonstrating normal ScvO₂ and CVP values at enrollment, thereby reducing the need for aggressive hemodynamic intervention. Earlier administration of appropriate antimicrobial therapy in the later studies may also have contributed to improved outcomes across all treatment groups and diminished the incremental benefit of EGDT. Additionally, differences in supportive therapies, including mechanical ventilation and inotropic use, likely reflect evolving hemodynamic phenotypes and changes in routine sepsis management practices [12]. Methodological considerations, including lower enrollment rates, high exclusion percentages, and the absence of blinding during inpatient management, may influence external validity, although the broader trend toward declining sepsis mortality remains consistent [12].

These findings contributed to a fundamental shift in sepsis resuscitation, moving away from rigid invasive protocols toward strategies emphasizing early recognition, prompt antimicrobial administration, and individualized fluid and vasopressor management, principles that now form the basis of contemporary SSC recommendations [3,10,11]. This transition was further reinforced by the Sepsis-3 framework, which emphasized identification of infection-associated organ dysfunction and recognition of sepsis as a heterogeneous syndrome requiring individualized clinical assessment rather than reliance on uniform protocolized targets [1].

Current sepsis management in the ED

Following the ProCESS, ARISE, and ProMISe trials, sepsis management in the ED has shifted from invasive, protocolized EGDT toward a streamlined, time-sensitive, and physiology-guided approach. Contemporary management emphasizes early recognition, prompt antimicrobial administration, and targeted hemodynamic stabilization rather than strict adherence to static physiologic targets [1-3,6,7,12]. The 2021 SSC Hour-1 Bundle remains a cornerstone of early sepsis management and outlines critical interventions that should be initiated as soon as sepsis or septic shock is recognized [3]. Current SSC recommendations are summarized in Table 4. Although strict completion of all interventions within one hour remains debated, the underlying principle that earlier intervention improves outcomes is widely accepted. 

**Table 4:** Current Surviving Sepsis Campaign Recommendations MAP: mean arterial pressure, CVP: central venous pressure. Data from References [3,13].

Intervention	Current Recommendation
Lactate measurement	Measure serum lactate and remeasure if initially elevated
Blood cultures	Obtain before antibiotics if this does not delay therapy
Antibiotics	Within 1 hour for septic shock/probable sepsis; within 3 hours after evaluation in selected patients without shock
Fluid resuscitation	Initial 30 mL/kg balanced crystalloid for hypotension or lactate ≥4 mmol/L
Vasopressors	Norepinephrine first-line; target MAP ≥65 mmHg
Fluid assessment	Dynamic measures preferred over static measures such as CVP
Perfusion monitoring	Lactate clearance and/or capillary refill time

Measure Lactate Levels

Serum lactate measurement serves as an important marker of tissue hypoperfusion and systemic metabolic stress. Consistent with Sepsis-3, elevated lactate levels are particularly important because they contribute to the identification of septic shock when present alongside persistent hypotension requiring vasopressor support despite adequate fluid resuscitation [1]. Lactate levels ≥4 mmol/L identify patients at increased risk who may require aggressive resuscitative measures, while serial lactate measurements provide valuable information regarding response to therapy [3]. Elevated lactate levels assist in determining both the urgency and intensity of resuscitation, facilitating a more individualized approach to management rather than reliance on rigid physiologic targets [1,12].

Obtain Blood Cultures Prior to Antibiotics

Blood cultures should be obtained promptly to facilitate identification of causative pathogens and guide subsequent targeted antimicrobial therapy. However, culture acquisition should not substantially delay antibiotic administration. Early culture collection improves diagnostic accuracy and supports antimicrobial stewardship by allowing later de-escalation of empiric therapy when appropriate [3].

Administer Broad-Spectrum Antibiotics

Timely antibiotic administration remains among the most important determinants of survival in patients with sepsis, particularly in septic shock. Initial empiric therapy should provide adequate coverage for likely pathogens based on the suspected source of infection, patient comorbidities, and local antimicrobial resistance patterns [3,12]. ED workflows increasingly incorporate rapid triage systems, sepsis alerts, and pharmacy-supported protocols to reduce time to antibiotic administration. Common empiric broad-spectrum antibiotic regimens include piperacillin-tazobactam, cefepime, or meropenem, selected according to the suspected source of infection, local antimicrobial resistance patterns, and patient-specific risk factors. Vancomycin should be added when methicillin-resistant *Staphylococcus aureus *(MRSA) infection is suspected or when patients have risk factors for MRSA colonization or infection [3,13].

The updated 2026 SSC guidelines further refined recommendations regarding antimicrobial timing. For patients with septic shock or probable/definite sepsis, immediate antibiotic administration, ideally within one hour, remains recommended. However, for patients with possible sepsis without shock, the guidelines support a brief period of focused diagnostic evaluation, with antimicrobial therapy initiated within three hours if concern for infection persists. This approach attempts to balance timely treatment with antimicrobial stewardship principles while minimizing unnecessary exposure to broad-spectrum agents [13].

Initiate Rapid Crystalloid Resuscitation for Hypotension or Elevated Lactate

Current recommendations support an initial bolus of 30 mL/kg of balanced crystalloid solutions, such as Lactated Ringer’s solution or Plasma-Lyte, for patients with hypotension or lactate levels ≥4 mmol/L [3,13-15]. Although this remains the standard initial approach, increasing evidence favors individualized fluid strategies guided by dynamic physiologic assessment to minimize risks associated with excessive fluid administration, including pulmonary edema and organ dysfunction [12,16,17].

Early Vasopressor Support to Maintain MAP ≥65 mmHg

Norepinephrine remains the preferred first-line vasopressor for patients with persistent hypotension despite fluid resuscitation. Emerging evidence suggests that earlier initiation of vasopressor therapy, in some cases concurrently with fluid administration, may improve tissue perfusion while reducing the complications associated with excessive fluid administration [3,12].

Dynamic Assessment of Fluid Responsiveness

Contemporary post-EGDT practice has shifted away from static preload markers such as CVP toward dynamic methods for assessing fluid responsiveness [12]. These techniques include passive leg raising (PLR), point-of-care ultrasound (POCUS) assessment of inferior vena cava variability, left ventricular outflow tract velocity-time integral changes, stroke volume variation, and pulse pressure variation in mechanically ventilated patients [3,12]. Additionally, the 2026 SSC guidelines suggest capillary refill time (CRT) as an adjunct marker of tissue perfusion that may complement lactate monitoring and hemodynamic assessment [13]. Such approaches allow real-time evaluation of physiologic response, minimizing unnecessary fluid administration and promoting patient-specific care.

Overall, modern sepsis management in the ED prioritizes rapid, individualized intervention over rigid protocol adherence. Key differences between the EGDT era and contemporary ED management are summarized in Table 5. Early recognition, prompt antimicrobial administration, dynamic fluid assessment, and judicious vasopressor use collectively define current best practice. The post-EGDT era reflects a broader understanding that outcomes in sepsis are primarily driven by timely, patient-centered resuscitation strategies rather than fixed hemodynamic targets alone [2,3,6,7,12].

**Table 5:** EGDT vs Modern Management EGDT: early goal-directed therapy, CVP: central venous pressure, ScvO₂: central venous oxygen saturation, MAP: mean arterial pressure, POCUS: point-of-care ultrasound, SVV: stroke volume variation, PPV: pulse pressure variation, ICU: intensive care unit. Data from References [2,3,5-7,12,13].

Feature	Early Goal-Directed Therapy (EGDT) Era	Contemporary Emergency Department Management
Overall approach	Protocolized, target-driven 6-hour resuscitation bundle	Individualized, physiology-guided, time-sensitive resuscitation
Hemodynamic assessment	Routine use of CVP and ScvO₂ monitoring via a central venous catheter	Dynamic assessment of fluid responsiveness using passive leg raising, POCUS, SVV, PPV, and capillary refill time
Resuscitation targets	CVP 8–12 mmHg, MAP ≥65 mmHg, ScvO₂ ≥70%	Focus on tissue perfusion, lactate clearance, capillary refill, and individualized hemodynamic goals
Fluid resuscitation	Standardized fluid administration to achieve protocol targets	Initial crystalloid resuscitation followed by individualized fluid administration guided by dynamic assessment
Vasopressor therapy	Typically initiated after completion of fluid resuscitation if hypotension persisted	Earlier norepinephrine initiation, often during ongoing fluid resuscitation when indicated
Antimicrobial therapy	Important component of care but less emphasized within original EGDT protocol	Early administration remains a cornerstone of management
Monitoring strategy	Routine invasive hemodynamic monitoring	Reduced reliance on invasive monitoring; greater use of bedside ultrasound and noninvasive physiologic assessment
Disposition	Frequent ICU admission for protocol implementation	Disposition based on illness severity and resource requirements
Primary goal	Achievement of predefined physiologic targets	Early recognition, rapid treatment, restoration of perfusion, and individualized patient-centered care

Controversies and evolving practices

Despite substantial advances in ED sepsis management, several aspects of care remain controversial. Major controversies and evolving areas of practice are summarized in Table 6. The transition from protocolized EGDT toward individualized resuscitation strategies, together with growing recognition of sepsis as a heterogeneous syndrome, has generated ongoing debate regarding optimal antimicrobial timing, fluid administration, perfusion targets, and biomarker-guided management [1,12]. These controversies reflect the challenge of balancing standardized evidence-based recommendations with patient-specific physiologic differences. Understanding these evolving areas of uncertainty is important for optimizing outcomes while minimizing unintended consequences.

**Table 6:** Current Controversies in Sepsis Management ScvO₂: central venous oxygen saturation. Data from References [3,12-22].

Controversy	Traditional Approach	Emerging Evidence
Antibiotic timing	Universal administration within 1 hour	Immediate treatment for shock; individualized approach for stable patients
Fluid resuscitation	Liberal fluid administration	Individualized fluid administration
Perfusion target	ScvO₂ monitoring	Lactate clearance and dynamic assessment of tissue perfusion
Vasopressor timing	After fluid completion	Earlier norepinephrine initiation
Crystalloid choice	Normal saline acceptable	Preference for balanced crystalloids
Biomarker use	Limited role	Increasing role in antimicrobial stewardship and personalized care

Timing of Antibiotic Administration: Is <1 Hour Always Necessary?

The SSC recommends administration of antibiotics within one hour of recognition, particularly in patients with septic shock, as delays in this population are consistently associated with increased mortality [3]. However, evidence supporting a universal one-hour mandate is less definitive in hemodynamically stable patients with suspected sepsis, raising concerns regarding overtreatment and inappropriate antimicrobial use. Critics argue that indiscriminate early administration of broad-spectrum agents may increase unnecessary exposure, contribute to antimicrobial resistance, and lead to avoidable adverse events [12].

Supporting these concerns, a multicenter retrospective cohort study involving 600 ED patients treated for suspected sepsis found that approximately one-third of patients likely did not have a bacterial infection, while four out of five patients with confirmed infections received antibiotic regimens that were broader than necessary in retrospect. Antibiotic-associated complications, including infections with resistant organisms, occurred in 17% of patients [18].

Complementing these findings, a systematic review and meta-analysis involving 79,246 patients demonstrated that antibiotic administration within one hour significantly reduced mortality only in patients with septic shock, whereas administration within one hour did not significantly affect mortality among patients with sepsis without shock. Importantly, antibiotic initiation within three hours reduced mortality in both septic shock and overall sepsis populations [19].

Collectively, these findings support risk-stratified antimicrobial strategies, whereby immediate therapy is prioritized for patients with septic shock or other high-risk features, while hemodynamically stable patients may undergo a brief period of focused diagnostic evaluation to optimize antimicrobial selection and reduce unnecessary treatment. This approach balances the urgency of sepsis management with antimicrobial stewardship principles and supports individualized patient care rather than strict adherence to a fixed temporal threshold. Updated recommendations similarly favor individualized, risk-based decision-making strategies [13].

Fluid Resuscitation: Liberal Versus Restricted Strategies

Current SSC guidelines recommend initial intravenous crystalloid administration, typically 30 mL/kg, for patients with septic shock to restore intravascular volume and optimize tissue perfusion [3,16]. Although early fluid administration remains essential, the optimal strategy following initial resuscitation remains uncertain. Both observational studies and randomized trials have raised concerns regarding the potential harms associated with excessive fluid administration, including pulmonary edema, fluid overload, and organ dysfunction [16].

The Crystalloid Liberal or Vasopressors Early Resuscitation in Sepsis (CLOVERS) trial, a multicenter randomized study involving 1,563 adults with sepsis-induced hypotension, compared a restrictive fluid strategy, emphasizing earlier vasopressor use with lower fluid volumes, with a liberal strategy involving greater fluid administration prior to vasopressor initiation [17]. Patients in the restrictive group received a median of 1,267 mL of fluid compared with 3,400 mL in the liberal group and received vasopressors earlier and more frequently. Despite these differences, 90-day mortality prior to discharge home was similar between groups (14.0% vs. 14.9%; P = 0.61), and serious adverse events were comparable. Furthermore, subgroup analyses did not identify patient populations preferentially benefiting from either strategy.

These findings suggest that among patients with sepsis-induced hypotension who have already received initial fluid resuscitation, a vasopressor-predominant strategy and a fluid-predominant strategy yield comparable outcomes [17]. Consequently, current practice increasingly emphasizes individualized fluid administration guided by hemodynamic monitoring, perfusion markers, and patient comorbidities rather than fixed-volume approaches.

Lactate Clearance Versus ScvO₂ Monitoring

EGDT originally emphasized central venous oxygen saturation (ScvO₂) as a primary resuscitation target, requiring invasive central venous catheter placement [5,12]. However, subsequent large randomized trials demonstrated no significant survival benefit associated with ScvO₂-targeted therapy, leading to increased reliance on lactate clearance as a marker of tissue perfusion and treatment response [12].

Lactate measurement offers several practical advantages, including widespread availability, minimal invasiveness, and substantial prognostic value, making it a preferred tool for resuscitation guidance in emergency settings. Although ScvO₂ monitoring may retain utility in selected refractory cases, lactate clearance has largely replaced it as a principal physiologic target during early sepsis management.

Supporting this transition, a retrospective study involving 9,190 patients with sepsis and intermediate lactate levels (2-4 mmol/L) demonstrated substantially lower hospital and 30-day mortality among patients achieving lactate clearance within 12 hours (8.2% vs. 18.7% and 13.3% vs. 24.7%, respectively). Additionally, each 10% increase in lactate was associated with a 9.4% increase in odds of hospital mortality [19]. The study also found that early fluid administration below 45 mL/kg modestly improved lactate clearance, further linking resuscitation strategy with physiologic outcomes.

Similarly, a 2018 meta-analysis of 16 randomized controlled trials comparing EGDT, lactate-guided therapy (LGT), and usual care found that LGT was associated with lower mortality than EGDT without increasing requirements for blood transfusions, vasopressors, or dobutamine [20]. These findings support lactate clearance as a practical and prognostically useful target that aligns with individualized, physiology-guided resuscitation strategies.

Early Vasopressor Use and Fluid Management in Sepsis-Induced Hypotension

Traditional sepsis protocols advocated completion of fluid resuscitation before vasopressor initiation. More recent evidence, however, supports earlier norepinephrine administration, sometimes concurrently with fluid therapy, to maintain perfusion pressure while minimizing complications associated with excessive fluid administration, including pulmonary edema, acute respiratory distress syndrome, and renal injury [3,12].

Norepinephrine remains the preferred first-line vasopressor, and the 2026 SSC guidelines support early initiation to maintain a target MAP of approximately 65 mmHg. Importantly, vasopressor initiation should not be unnecessarily delayed while awaiting completion of fluid administration [13].

Beyond fluid volume, fluid composition has also emerged as an important consideration. The SMART trial demonstrated that among critically ill adults, balanced crystalloids reduced the composite outcome of death, new renal replacement therapy, or persistent renal dysfunction compared with normal saline (14.3% vs. 15.4%; marginal OR 0.91, 95% CI 0.84-0.99; P = 0.04). Benefits were particularly pronounced among patients with sepsis, with a reduction in 30-day mortality from 29.4% to 25.2% (adjusted OR 0.80, 95% CI 0.67-0.97; P = 0.02) [14].

Similarly, the SALT-ED trial found that balanced crystalloids reduced major adverse kidney events within 30 days compared with saline (4.7% vs. 5.6%; adjusted OR 0.82, 95% CI 0.70-0.95; P = 0.01) among non-critically ill ED patients [15]. These findings support preferential use of balanced crystalloids in routine ED resuscitation.

Overall, current evidence suggests that following initial resuscitation, clinicians should moderate ongoing fluid administration, favor balanced crystalloid solutions, and consider earlier vasopressor use while tailoring treatment according to individual physiologic characteristics [12,14,15,17].

Biomarkers and Personalized Sepsis Care

Sepsis represents a highly heterogeneous syndrome, and biomarkers are increasingly used to refine diagnosis and guide management. This heterogeneity was specifically recognized by the Sepsis-3 task force, which emphasized that sepsis encompasses a broad spectrum of clinical and biological phenotypes influenced by host factors, comorbidities, pathogen characteristics, and variations in the immune response [1]. Conventional biomarkers, including procalcitonin (PCT) and C-reactive protein (CRP), may assist antimicrobial stewardship efforts but lack sufficient specificity for definitive diagnosis. Lactate remains central to risk stratification and resuscitation guidance.

Emerging biomarkers, including presepsin, cytokine profiling, and machine learning-based predictive models, demonstrate potential for identifying biologically distinct sepsis subtypes and facilitating personalized treatment approaches. Gene-expression-derived subphenotypes such as Sepsis Response Signatures (SRS1 and SRS2) have been validated across bacterial and viral sepsis populations and exhibit distinct immunologic characteristics and prognostic profiles [21].

Despite their promise, the routine implementation of emerging biomarkers in ED practice remains limited by several practical considerations. High costs, limited availability, variable turnaround times, and the need for specialized laboratory infrastructure may restrict their widespread adoption, particularly in resource-constrained settings. Furthermore, many novel biomarkers require additional validation before they can be routinely incorporated into clinical decision-making and international sepsis management guidelines [21].

The ADAPT-Sepsis randomized clinical trial further supported the use of biomarker-guided treatment strategies in hospitalized patients with suspected sepsis [22]. In this multicenter study involving 2,760 critically ill patients, PCT-guided protocols reduced antibiotic duration while maintaining noninferior 28-day mortality. In contrast, CRP-guided protocols did not significantly reduce antibiotic duration and yielded inconclusive mortality findings.

These findings support the role of biomarkers in improving antimicrobial stewardship and reducing over-treatment. Nevertheless, widespread implementation remains limited by cost, accessibility, and lack of standardized thresholds [12]. As understanding of sepsis heterogeneity continues to evolve, biomarker-guided and phenotype-based approaches may facilitate more individualized treatment strategies in the future. However, these technologies should complement rather than replace careful clinical assessment and physiologic monitoring in the ED.

ED-specific considerations

Triage and Early Sepsis Screening Tools

Early identification of sepsis during triage is essential for improving patient outcomes and initiating timely interventions. Because sepsis is a time-sensitive syndrome associated with increasing mortality as organ dysfunction progresses, several bedside screening and risk-stratification tools have been developed to facilitate rapid recognition in the ED [1].

The qSOFA score incorporates three criteria: respiratory rate ≥22 breaths/min, systolic blood pressure ≤100 mmHg, and altered mental status. The Sepsis-3 task force introduced qSOFA as a rapid bedside tool intended to identify patients with suspected infection who may be at increased risk of adverse outcomes outside intensive care settings [1]. Although qSOFA is simple and rapidly applied, its relatively low sensitivity limits its usefulness as a primary screening tool, and it is more appropriately used as a prognostic indicator rather than a diagnostic screening instrument for sepsis itself.

Compared with qSOFA, broader early warning scores such as the National Early Warning Score 2 (NEWS2) incorporate additional physiological parameters, including heart rate, oxygen saturation, temperature, and supplemental oxygen requirements, resulting in greater sensitivity for the early identification of clinical deterioration and sepsis. However, this improved sensitivity may come at the expense of lower specificity and increased false-positive alerts. Consequently, NEWS2 is generally preferred for early screening, whereas qSOFA is better suited for identifying patients at higher risk of adverse outcomes [13].

The 2026 SSC guidelines recommend broader physiologic scoring systems, including NEWS, NEWS2, Modified Early Warning Score (MEWS), and Systemic Inflammatory Response Syndrome (SIRS) criteria, over the use of qSOFA alone because of the latter's relatively limited sensitivity for early sepsis detection. Nevertheless, no single scoring system should replace clinical judgment [13,23].

Importantly, no individual screening tool can reliably identify all patients with sepsis. Consistent with the Sepsis-3 framework, sepsis should ultimately be viewed as a clinical syndrome characterized by infection-associated organ dysfunction rather than by fulfillment of a specific screening score alone [1]. The Sepsis-3 definition emphasizes organ dysfunction in the setting of infection, quantified by a SOFA score increase of ≥2 points, while septic shock is defined by persistent hypotension requiring vasopressor support to maintain a MAP ≥65 mmHg with lactate levels >2 mmol/L despite adequate fluid resuscitation. In contrast, the CMS SEP-1 criteria define septic shock as systolic blood pressure <90 mmHg or lactate ≥4 mmol/L, without requiring vasopressor administration. Consequently, early recognition should focus on suspected infection combined with evidence of organ dysfunction or elevated lactate levels, regardless of whether formal screening tools are triggered [4].

Emerging artificial intelligence and machine learning technologies may further improve early detection in the ED by identifying subtle clinical patterns and high-risk presentations that conventional scoring systems may fail to recognize [4]. Such approaches may help address one of the central challenges highlighted by Sepsis-3: the substantial clinical heterogeneity of patients presenting with sepsis [1].

Challenges in Low-Resource or High-Volume EDs

Implementation of sepsis screening tools and care pathways presents unique challenges in resource-limited and overcrowded EDs. Staffing limitations may delay patient assessment and recognition of sepsis, while the restricted availability of point-of-care lactate testing can hinder rapid risk stratification. High patient volumes may also reduce attention to subtle presentations of early sepsis, and infrastructure limitations, such as the absence of electronic alert systems or limited monitoring equipment, may impair adherence to established protocols.

These barriers highlight the need for pragmatic and context-specific adaptations of sepsis pathways that balance adherence to evidence-based guidelines with local operational realities and available resources [23].

Importance of Multidisciplinary Sepsis Pathways

Effective sepsis management requires coordinated involvement of multiple healthcare disciplines. Emergency physicians and nursing staff are primarily responsible for early recognition and initial resuscitation, while pharmacists facilitate timely antimicrobial delivery. Critical care teams provide advanced support for patients requiring escalation of care, and laboratory and radiology services contribute to rapid diagnostic evaluation.

Multidisciplinary pathways, frequently implemented as structured sepsis bundles, improve coordination of care and reduce practice variability [23]. Evidence supports prompt administration of intravenous crystalloid therapy, typically using an initial 30 mL/kg bolus for patients with hypotension or evidence of tissue hypoperfusion. However, resuscitation strategies should remain individualized according to patient characteristics and physiologic response. In certain settings, assessment of peripheral perfusion may demonstrate outcomes comparable to lactate-guided resuscitation. Fluid administration should also be tailored in special populations, including patients with obesity, in whom adjusted body weight may be used for volume calculations [4].

Updated recommendations further emphasize the role of structured institutional pathways, including multidisciplinary “Code Sepsis” systems designed to facilitate rapid recognition and implementation of time-sensitive interventions [13].

Integration of Sepsis Protocols into Electronic Health Records

Integration of sepsis protocols into electronic health record (EHR) systems can improve standardization and adherence to evidence-based practices. Automated alerts may identify patients meeting sepsis criteria, while standardized order sets can facilitate rapid implementation of the Hour-1 bundle. Clinical decision support systems may further assist with fluid management, vasopressor selection, and antimicrobial prescribing, while integrated data monitoring enables continuous quality assessment and performance improvement initiatives.

By translating guideline recommendations into real-time clinical workflows, EHR integration may help bridge the gap between evidence and practice, particularly in high-volume EDs where reliance on manual recognition alone may be insufficient [23].

Regional considerations: the Gulf region and Bahrain

Published data regarding sepsis and severe bloodstream infections in the Gulf region remain limited and are largely derived from single-center, intensive care unit-based studies, highlighting the need for broader regional surveillance systems and multicenter research initiatives. Consistent application of standardized sepsis definitions, such as those proposed by the Sepsis-3 task force, may improve comparability between regional studies and facilitate more accurate assessment of disease burden and outcomes across healthcare systems [1]. The existing literature from Gulf Cooperation Council (GCC) countries has documented increasing challenges posed by multidrug-resistant organisms, including high rates of carbapenem-resistant Gram-negative pathogens, which may complicate empiric antimicrobial selection and contribute to poorer clinical outcomes [24]. Among the most frequently encountered resistant Gram-negative organisms in the region are *Escherichia coli *and *Klebsiella pneumoniae*, with widespread reports of extended-spectrum β-lactamase (ESBL) production and the emergence of carbapenemase genes such as *NDM *and *OXA-48*, emphasizing the importance of selecting empiric antimicrobial therapy according to local resistance patterns and institutional antibiograms [25].

The growing burden of antimicrobial resistance has important implications for ED management, where empiric antibiotic therapy frequently precedes microbiological confirmation. Regional variations in pathogen distribution and resistance profiles underscore the importance of developing locally adapted antimicrobial protocols rather than relying exclusively on international recommendations. In many Gulf healthcare settings, broad-spectrum β-lactam/β-lactamase inhibitor combinations, such as piperacillin-tazobactam, remain appropriate empiric therapy for many patients with suspected sepsis; however, patients with risk factors for ESBL-producing or carbapenem-resistant organisms may require broader initial coverage guided by local susceptibility data and antimicrobial stewardship principles [24,25]. Data specific to Bahrain remain similarly limited, emphasizing the need for national epidemiologic surveillance, antimicrobial stewardship initiatives, and standardized sepsis management pathways tailored to local healthcare settings [24].

Improved regional data collection, standardized application of contemporary sepsis definitions, and collaborative quality-improvement efforts may strengthen evidence-based sepsis management strategies and enhance the applicability of international guidelines to Gulf populations [1,24].

Limitations

This review has several limitations. Included studies differed substantially in methodology, patient populations, clinical settings, and evolving definitions of sepsis across different time periods. In particular, changes in sepsis definitions from systemic inflammatory response syndrome (SIRS)-based criteria to the Sepsis-3 framework may affect the interpretation and comparison of findings across studies conducted in different eras. Variability in study design, patient selection, and outcome measures may further limit direct comparisons between investigations.

Additionally, many landmark trials and guideline recommendations were developed within high-resource healthcare systems, potentially limiting generalizability to resource-constrained environments and regional Gulf populations. Differences in healthcare infrastructure, patient demographics, pathogen distribution, and antimicrobial resistance patterns may further influence the applicability of published evidence.

As a narrative review, this article is also subject to the inherent limitations of non-systematic literature synthesis, including the potential for selection bias and reliance on the available published literature. Although priority was given to landmark trials, major guidelines, systematic reviews, and meta-analyses, relevant studies may not have been included.

Finally, sepsis management continues to evolve rapidly, and emerging evidence may alter current recommendations, particularly in areas such as biomarker-guided therapy, precision medicine, artificial intelligence-assisted clinical decision support, and phenotype-based approaches to sepsis care. Consequently, some conclusions presented in this review may require reassessment as future evidence becomes available.

## Conclusions

Sepsis remains a major cause of morbidity and mortality worldwide, making early recognition and timely intervention essential in the ED. Over the past two decades, sepsis management has evolved substantially from the protocolized approach of EGDT toward individualized, physiology-guided resuscitation strategies. Although subsequent trials demonstrated that rigid hemodynamic targets do not provide additional benefit over contemporary standard care, the core principles introduced by EGDT, i.e., early diagnosis, prompt antimicrobial therapy, and aggressive initial resuscitation, continue to underpin modern sepsis management.

Current practice emphasizes rapid diagnosis, appropriate antimicrobial administration, balanced crystalloid resuscitation, dynamic assessment of fluid responsiveness, and early vasopressor support when indicated. Future advances are expected to focus on biomarker-guided management, improved identification of sepsis phenotypes, precision medicine approaches, and artificial intelligence-assisted clinical decision support. Continued integration of these developments with evidence-based care pathways may further improve outcomes and support more personalized management of sepsis in the ED.

## References

[1] Singer M, Deutschman CS, Seymour CW (2016). The third international consensus definitions for sepsis and septic shock (sepsis-3). JAMA.

[2] Yealy DM, Kellum JA, Huang DT (2014). A randomized trial of protocol-based care for early septic shock. N Engl J Med.

[3] Evans L, Rhodes A, Alhazzani W (2021). Surviving sepsis campaign: international guidelines for management of sepsis and septic shock 2021. Intensive Care Med.

[4] Hwang EH, Hwang CW, Augustin B, Guirgis FW, Black LP (2025). Updates and controversies in the early management of sepsis and septic shock. Emerg Med Pract.

[5] Rivers E, Nguyen B, Havstad S (2001). Early goal-directed therapy in the treatment of severe sepsis and septic shock. N Engl J Med.

[6] Peake SL, Delaney A, Bailey M (2014). Goal-directed resuscitation for patients with early septic shock. N Engl J Med.

[7] Mouncey PR, Osborn TM, Power GS (2015). Trial of early, goal-directed resuscitation for septic shock. N Engl J Med.

[8] Purcarea A, Sovaila S (2020). Sepsis, a 2020 review for the internist. Rom J Intern Med.

[9] Gavelli F, Castello LM, Avanzi GC (2021). Management of sepsis and septic shock in the emergency department. Intern Emerg Med.

[10] Dellinger RP, Levy MM, Rhodes A (2013). Surviving Sepsis Campaign: international guidelines for management of severe sepsis and septic shock, 2012. Intensive Care Med.

[11] Zhang Z, Hong Y, Smischney NJ (2017). Early management of sepsis with emphasis on early goal directed therapy: AME evidence series 002. J Thorac Dis.

[12] Nguyen HB, Jaehne AK, Jayaprakash N (2016). Early goal-directed therapy in severe sepsis and septic shock: insights and comparisons to ProCESS, ProMISe, and ARISE. Crit Care.

[13] Prescott HC, Antonelli M, Alhazzani W (2026). Surviving sepsis campaign: international guidelines for management of sepsis and septic shock 2026. Crit Care Med.

[14] Semler MW, Self WH, Wanderer JP (2018). Balanced crystalloids versus saline in critically Ill adults. N Engl J Med.

[15] Self WH, Semler MW, Wanderer JP (2018). Balanced crystalloids versus saline in noncritically ill adults. N Engl J Med.

[16] Meyhoff TS, Hjortrup PB, Wetterslev J (2022). Restriction of intravenous fluid in ICU patients with septic shock. N Engl J Med.

[17] Shapiro NI, Douglas IS, Brower RG (2023). Early restrictive or liberal fluid management for sepsis-induced hypotension. N Engl J Med.

[18] Shappell CN, Yu T, Klompas M (2025). Frequency of antibiotic overtreatment and associated harms in patients presenting with suspected sepsis to the emergency department: a retrospective cohort study. Clin Infect Dis.

[19] Liu V, Morehouse JW, Soule J, Whippy A, Escobar GJ (2013). Fluid volume, lactate values, and mortality in sepsis patients with intermediate lactate values. Ann Am Thorac Soc.

[20] Ding XF, Yang ZY, Xu ZT (2018). Early goal-directed and lactate-guided therapy in adult patients with severe sepsis and septic shock: a meta-analysis of randomized controlled trials. J Transl Med.

[21] Antcliffe DB, Burrell A, Boyle AJ, Gordon AC, McAuley DF, Silversides J (2025). Sepsis subphenotypes, theragnostics and personalized sepsis care. Intensive Care Med.

[22] Dark P, Hossain A, McAuley DF (2025). Biomarker-guided antibiotic duration for hospitalized patients with suspected sepsis: the ADAPT-Sepsis randomized clinical trial. JAMA.

[23] Al-Musawi T, Al-Agha R, Al-Khiami S (2025). Bacteremia in the Gulf Cooperation Council region: a review of the literature 2013-2023. Infect Drug Resist.

[24] Carvey MM, Glauser J (2025). The management of severe sepsis and septic shock: a novel update and bedside reference guide. Curr Emerg Hosp Med Rep.

[25] Bindayna KM, Joji RM, Ezzat H (2022). Antibiotic-resistance genes in Escherichia coli strains in GCC countries: a meta-analysis. J Fam Community Med.

